# Effect of folate intake on health outcomes in pregnancy: a systematic review and meta-analysis on birth weight, placental weight and length of gestation

**DOI:** 10.1186/1475-2891-11-75

**Published:** 2012-09-19

**Authors:** Katalin Fekete, Cristiana Berti, Monica Trovato, Szimonetta Lohner, Carla Dullemeijer, Olga W Souverein, Irene Cetin, Tamás Decsi

**Affiliations:** 1 Department of Biochemistry and Medical Chemistry, University of Pécs, Szigeti út 12, H-7624, Pécs, Hungary; 2 Unit of Obstetrics and Gynecology and Center for Fetal Research Giorgio Pardi, University of Milan, Milan, Italy; 3 Department of Paediatrics, University of Pécs, Pécs, Hungary; 4 Division of Human Nutrition, Wageningen University and Research Centre, Wageningen, The Netherlands

**Keywords:** Folate/folic acid, Pregnancy, Birth weight, Placental weight, Length of gestation, EURRECA

## Abstract

The beneficial effect of folic acid supplementation before and shortly after conception is well recognized, whereas the effect of supplementation during the second and third trimesters is controversial and poorly documented. Our aims were to systematically review randomized controlled trials (RCTs) investigating the effect of folate supplementation on birth weight, placental weight and length of gestation and to assess the dose–response relationship between folate intake (folic acid plus dietary folate) and health outcomes. The MEDLINE, EMBASE and Cochrane Library CENTRAL databases were searched from inception to February 2010 for RCTs in which folate intake and health outcomes in pregnancy were investigated. We calculated the overall intake-health regression coefficient ( $\hat{\beta }$) by using random-effects meta-analysis on a log_e_-log_e_ scale. Data of 10 studies from 8 RCTs were analyzed. We found significant dose–response relationship between folate intake and birth weight (P=0.001), the overall $\hat{\beta }$ was 0.03 (95% confidence interval (CI): 0.01, 0.05). This relationship indicated 2% increase in birth weight for every two-fold increase in folate intake. In contrast, we did not find any beneficial effect of folate supplementation on placental weight or on length of gestation. There is a paucity of well-conducted RCTs investigating the effect of folate supplementation on health outcomes in pregnancy. The dose–response methodology outlined in the present systematic review may be useful for designing clinical studies on folate supplementation and for developing recommendations for pregnant women.

## Introduction

Folate plays a crucial role in the one-carbon metabolism for physiological nucleic acid synthesis and cell division, regulation of gene expression, amino acid metabolism and neurotransmitter synthesis [1]. During pregnancy, increased folate intake is required for rapid cell proliferation and tissue growth of the uterus and the placenta, growth of the fetus and expansion of the maternal blood volume [2]. Folate requirements are 5- to 10-fold higher in pregnant than in non-pregnant women [3], therefore pregnant women may be at risk for folate deficiency.

The importance of adequate periconceptional folate supply is well recognized in human health; the link between maternal folate status and fetal neural tube defects (NTDs) [4,5] and other congenital malformations [6-10] is generally accepted. In most countries women are advised to use folic acid supplements in the periconceptional period: 0.4 mg per day when planning a pregnancy, or 4 mg per day when a previous pregnancy was affected by NTD [11].

However, the effect of folate supplementation throughout pregnancy on several other health outcomes is highly controversial [12]. Numerous observational studies suggest a potential benefit of good maternal folate status on birth weight, placental weight or length of gestation [13-18]. In contrast, supplementation trials have shown equivocal results: some supplementation trials reported no effect [19-24] whereas other trials reported significant beneficial effect of folate supplementation on at least one of the above-mentioned pregnancy outcomes [25-28].

The aims of this systematic review and meta-analysis were to summarize the evidence from randomized controlled trials (RCTs) and to assess the dose–response relationship between folate intake and birth weight, placental weight as well as length of gestation.

## Methods

The research presented here is part of a project within the European Micronutrient Recommendations Aligned (EURRECA) network that aims to identify micronutrient requirements for optimal health in European populations ( http://www.eurreca.org). Data reported in this systematic review represent part of a wider review process aimed to identify studies assessing the effect of folate intake on different markers of folate status and health outcomes in different population subsets with potential folate deficiency.

### Search strategy

Electronic searches were carried out in Ovid MEDLINE ( http://www.ovid.com), EMBASE (Ovid) ( http://www.ovid.com) and the Cochrane Library CENTRAL database ( http://www.thecochranelibrary.com) from inception to February 2010. Text terms with appropriate truncation and indexing terms were used to identify articles eligible for review. The general search strategy was in the following form: [randomized controlled trials] AND [human studies] AND [intake or status] AND [folate or folic acid or vitamin B_9_]. The search strategy was adapted to each of the individual databases. The electronic searches were supplemented with hand searches of journals, and the reference lists from relevant articles located were used to identify additional sources. We did not apply any language restriction.

### Inclusion criteria

To be included, a study needed to meet the following criteria: 1. supplementation study in healthy pregnant women, 2. RCT with control group which received placebo or no treatment (in the case of combined intervention with other micronutrients the only difference between intervention and control group is folate supplementation), 3. minimum duration of supplementation of 12 weeks, 4. report folate intake from supplements, fortified foods or natural food sources, and 5. report one or more of the following health outcomes: birth weight, placental weight and length of gestation. Only papers meeting all inclusion criteria were included in the review.

### Data collection and extraction

Titles and abstracts were screened for inclusion by two independent reviewers, with duplicate assessment of a random sample of 10% in order to harmonize the process. The full text of the potentially relevant titles and abstracts was screened for inclusion by using an inclusion/exclusion form. Included papers were extracted into a Microsoft Access 2003 database file (Microsoft Corp, Redmond, WA) by two independent reviewers. The database included bibliographic and methodological details, population characteristics, intervention details as well as outcome data. In doubtful cases, studies were discussed within the review team before beginning full data extraction.

### Assessment of internal validity of included studies

In order to assess the risk of bias in the studies, the following indicators of internal validity specific to the RCT methodology were collected: 1. method of sequence generation and allocation, 2. blinding of participants, 3. number of participants at start, dropouts and dropout reasons, 4. funders, 5. compliance check, 6. dose check, 7. dietary intake data reported, 8. outcome comparability and reproducibility and 9. similarity of most and least exposed groups at baseline. The criteria for evaluating these indicators were adapted from the Cochrane Handbook [29]. Based on these indicators, two reviewers decided on the overall risk of bias.

### Data synthesis

When necessary, units of measurement were converted to a standard form to facilitate comparison across studies. If data were not presented in mean ± standard deviation, they were converted into this format using methods described in the Cochrane Handbook [29]. Taking into account that synthetic folic acid is more bioavailable than natural food folates, the amount of folic acid from supplements was transformed into amount of folate (multiplied by 1.7 to express it in Dietary Folate Equivalents) [30]. When dietary intake was not provided, the mean dietary folate intake (247 μg/day) from other comparable studies investigating the relationship between folate intake and status [31] was included in the calculation.

We calculated an intake-health regression coefficient ( $\hat{\beta }$) and its standard error (SE) for each individual study [32]. The intake-health relationship was assumed to be linear on the log_e_-log_e_ scale (natural logarithm of intake versus natural logarithm of the single health outcome). This assumption is based upon our hypothesis that the true intake-health curve for folate would follow a natural logarithmic function, slowly growing to positive infinity as x (intake) increases and rapidly going to negative infinity as x (intake) approaches 0. However, the true shape of the relationships that we investigated is mostly unknown. This shape of the curve (monotonic concave) is a likely shape in biology. Therefore, we used this assumption as a practical approximation. The basics of the statistical model underlying the meta-analysis are described in detail elsewhere [32].

Meta-analysis was carried out with Cochrane software, Review Manager Version 5 (Cochrane Collaboration; http://www.cochrane.org); we calculated the overall $\hat{\beta }$ using random-effects model. A statistically significant result indicated that the health outcome was indeed responding to supplementation. Levels of the heterogeneity were noted (heterogeneity was considered significant where P < 0.1 on the chi-square test, or I^2^ > 50%).

## Results

### Study inclusion

**Figure 1:**
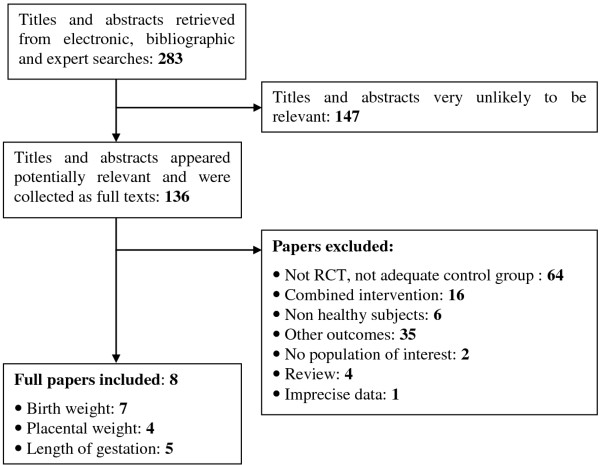
Flow diagram of the systematic literature search.

The flow diagram of the literature search for this systematic review is shown on Figure 1. Altogether 4067 hits were identified through the general electronic and bibliographic searches. A total of 3784 titles appeared to be inappropriate, only 283 described the population of pregnant women and appeared to be potentially relevant for the aim of this review. After abstract screening, 136 were collected as full-text papers and finally 8 publications fulfilled our inclusion criteria. One article contained two different ethnic groups [25] and one article contained two different interventions [24]. These data were analyzed each as two separate studies, giving a total of 10 substudies from 8 publications. The most common reasons for exclusion were inappropriate study design, combined intervention, i.e. multivitamin supplementation vs. placebo and not relevant study outcome. 

The characteristics of the studies included in the analysis are presented in Table 1. The age of pregnant women was reported only in three studies [19,23,24]; one of them included pregnant adolescents [23]. Three studies were carried out in Europe [24,28,33], two in South America [19,23], two in Asia [26,27] and one in Africa [25]. The supplement was synthetic folic acid in most cases, subjects were treated with 5-methyltetrahydrofolate (5-MTHF) in one study [24]. In those studies in which folic acid was used the intervention group received folic acid together with an iron supplement, while the control group received an iron supplement alone. 5-MTHF versus placebo and 5-MTHF together with fish oil versus fish oil were used in the study by Klingler et al. [24]. 

**Table 1:** Basic characteristics of included studies

**Study**	**Participant characteristics**			**Short description of intervention**	Total folate intake^*^(μg/day)	Mean duration of intervention^**^	**Health outcomes**
	**Country**	**Age**	**No. in intervention and control groups**				
Baumslag 1970 [25]	South Africa	N/A	65; 63^a^ and 62; 52^b^	200 mg iron + 5 mg FA/day	8620^a^; 8747^b^	16 wk^a^ and 12 wk^b^	birth weight
				200 mg iron/day	120^a^; 247^b^		
Iyengar 1971 [26]	India	N/A	23; 26	60 mg iron + 300 μg FA/day	757	18 wk	birth weight
				60 mg iron/day	247		
Iyengar 1975 [27]	India	N/A	98; 91	60 mg iron + 500 μg FA/day	1097	16 wk	birth weight; placental weight
				60 mg iron/day	247		
Klingler 2006 [24]	Spain	18-40 y	16; 12	400 μg 5-MTHF/day	647	20 wk	birth weight; placental weight; length of gestation
			11; 16	placebo/day	247		
				fish oil + 400 μg 5- MTHF/day	647		
				fish oil/day	247		
Lira 1989 [19]	Chile	26.8±4.3 y 26.6±5.3 y	75; 78	105 mg iron + 350 μg FA/day	842	24 wk	length of gestation
				105 mg iron/day	247		
Nogueira 2002 [23]	Brasil	13-18 y	15; 13	120 mg iron sulphate + 250 μg FA/day	545	22 wk	birth weight; length of gestation
				120 mg iron sulphate/day	120		
Rolschau 1979 [33]	Denmark	N/A	20; 16	250 mg ferrofumarate + 5 mg FA/day	8747	17 wk	birth weight; placental weight; length of gestation
				250 mg ferrofumarate/day	247		
Tchernia 1982 [28]	France	N/A	54; 54	iron + μg FA/day	842	12 wk	birth weight; placental weight; length of gestation
				iron/day	247		

Abbreviations: *a*, data of Bantu women; *b*, data of White women; *FA*, folic acid; *5-MTHF*, 5-methyltetrahydrofolate; *N/A*, data not available; *, expressed as Dietary Folate Equivalents; **, supplements were administered from the second trimester to delivery in each study.

Each included study represented high risk of bias (data not shown). There was complete absence of data regarding the method of randomization; the blinding was adequate only in one study [23], and the reasons for dropping out were reported in three studies only [19,24,33].

### Birth weight

Birth weight was the most frequently reported health outcome, it was measured in 9 studies from 7 publications including 707 participants. Studies used a wide range of supplementation doses from 0.25 mg to 5 mg folic acid per day. The duration of supplementation varied among studies from 12 to 22 weeks (Table 1).

**Figure 2:**
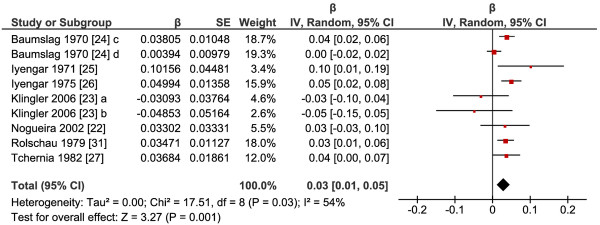
**Response of birth weight to supplementation with folic acid or 5-methyltetrahydrofolate.** Abbreviations: a, 5-methyltetrahydrofolate versus placebo; b, 5-methyltetrahydrofolate and with fish oil versus fish oil; c, Bantu women; d White women.

Folate supplementation resulted in significantly increased birth weight in the intervention group compared to the placebo group (P=0.001); the pooled effect estimate of the relationship between total folate intake (dietary folate plus folic acid/folate supplement) and birth weight was 0.03 (95% confidence interval (CI): 0.01, 0.05) (Figure 2). This means that two-fold increase in folate intake corresponds to a 1.02-fold (2^β^=2^0.03^) higher birth weight, which is 2% increase. The test for heterogeneity showed moderate heterogeneity (I^2^=54%) among studies. There were insufficient studies to perform further analysis of different subgroups according to supplement form, gestational age, dose or duration.

### Placental weight

Altogether 5 studies from 4 publications addressed the association between folate intake and placental weight; 199 participants in the intervention group and 189 participants in the control group were included. The duration of supplementation ranged from 12 to 20 weeks. The lowest supplementation dose was 0.35 mg, while the highest supplementation dose was 5 mg folic acid per day (Table 1).

**Figure 3:**
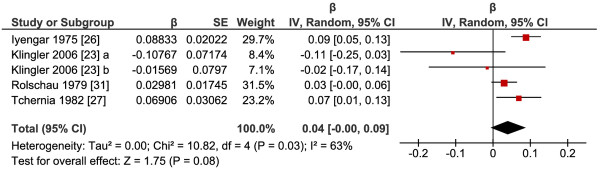
**Response of placental weight to supplementation with folic acid or 5-methyltetrahydrofolate.** Abbreviations: a, 5-methyltetrahydrofolate versus placebo; b, 5-methyltetrahydrofolate and with fish oil versus fish oil.

As it can be seen on Figure 3, the meta-analysis revealed no significant effect of total folate intake on placental weight (P=0.08), the pooled $\hat{\beta }$ was 0.04 (95% CI: 0.00, 0.09) with significant heterogeneity (I^2^=63%). The low number of studies available did not allow further analysis of different subgroups according to supplement form, gestational age, dose or duration.

### Length of gestation

Six studies from 5 publications involving 380 participants contained eligible data for the meta-analysis. Daily dose of folic acid supplementation varied from 0.25 mg to 5 mg, the duration was from 12 to 24 weeks (Table 1).

**Figure 4:**
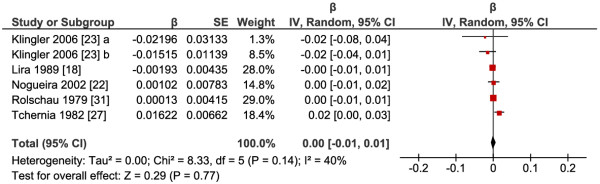
**Response of length of gestation to supplementation with folic acid or 5-methyltetrahydrofolate.** Abbreviations: a, 5-methyltetrahydrofolate versus placebo; b, 5-methyltetrahydrofolate and with fish oil versus fish oil.

The meta-analysis of the 6 RCTs available failed to show any significant effect of increased folate intake on length of gestation (P=0.77), the pooled $\hat{\beta }$ was 0.00 (95% CI: -0.01, 0.01) (Figure 4). There was low heterogeneity between studies (I^2^=40%). Like in the case of the previous health outcomes, we were not able to perform subgroup analysis.

## Discussion

Birth weight is one of the most important pregnancy outcome parameters; it is strongly associated with infant mortality during the first year of life and influences later developmental processes as well [34]. Folate required for growth reaches the maximal level in the last trimester [35], because of rapid growth of the fetus and the uteroplacental system and fetal accumulation of folate stores. Without sufficient folate intake, maternal plasma and red blood cell (RBC) folate decreases from the fifth month of pregnancy until several weeks after delivery [36]. A recent prospective study has also shown that low folate intake (< 187 μg/day) and low RBC folate status in the late pregnancy increase the risk of small for gestational age (SGA) birth in an adolescent population [37]. Other studies reported positive association between birth weight and maternal RBC folate status [13,38].

The present systematic review was aimed to summarize available data on the role of folate status in basic aspects of pregnancy outcome. Moreover, we included 10 studies (from 8 published papers) into a meta-analysis in order to assess whether there is any dose–response relationship between folate intake and birth weight, placental weight and length of gestation.

We applied base-e logarithmic transformation on folate intake and on the aforementioned pregnancy outcome parameters. These transformations make it possible to pool $\hat{\beta }$ values and report them as dose–response relationship between intake and health. The overall $\hat{\beta }$ represents the difference in the log_e_-transformed predicted value of the given health outcome for each one-unit difference in the log_e_-transformed value in folate intake. The intervention was started from the second trimester in all the included studies; therefore our results allow inferences about supplementation during pregnancy which differ substantially from periconceptional folate supplementation.

Our results support the hypothesis that increased folate intake after the first trimester is associated with higher birth weight. The overall $\hat{\beta }$ was found to be 0.03 indicating that a two-fold increase in folate intake corresponds to a 2% higher birth weight, which is a slight but significant increase. Or to put it in another way, a neonate whose mother has a folate intake of 500 μg per day is predicted to have a birth weight that is 2% higher than a neonate whose mother has a folate intake of 250 μg per day.

Placental weight is an important determinant of fetal weight. It has been also demonstrated that placental weight was significantly lower in SGA neonates compared to appropriate for gestational age neonates of the same birth weight [39]. Placental uptake of folate from the maternal circulation is critical for adequate folate supply to the developing fetus. Maternal folate is transferred against a concentration gradient to the fetus, the net effect is a two-fold higher plasma folate level of the neonates compared to the maternal plasma level at delivery [40]. Inadequate folate status during pregnancy may be a risk factor of several adverse health outcomes, such as fetal malformations and various placenta-related diseases [41]. Moreover, low folate status results in elevated plasma homocysteine level, which may increase the risk of placental damage and dysfunction, disturbing thereby oxygen and nutrient transport to the fetus [42].

In the present study we failed to detect any dose response relationship between folate intake and placental weight; our data did not show significantly elevated placental weight in treatment groups compared to placebo groups (P=0.08). In contrast to the growth of the fetus, the placenta grows rapidly in the first trimester and reaches its full size during the second trimester [43], therefore folate supplementation may have no further effect on placental weight in the later period of pregnancy.

A prospective study conducted on more than 2000 pregnant women demonstrated that low serum folate is associated with nearly a double risk of preterm delivery [44]. Scholl and colleagues have found similar degree of risk for preterm delivery in women with low folate intake (≤ 240 μg per day) [45]. Still, like in the case of placental weight, we did not find significant effect of folate supplementation on the length of gestation in the intervention groups compared to placebo groups (P=0.77).

The strength of our meta-analysis is the inclusion of RCTs. Ideally, RCTs should provide reliable data about the effect of an intervention. Randomization allows us to assume that changes in birth weight, placental weight or length of gestation are definitely due to folate intervention. Other factors that might affect these pregnancy outcomes would be expected to be distributed randomly between the intervention and control groups.

The findings of this meta-analysis must be interpreted in the light of certain limitations. First of all, the majority of studies were conducted at least 30 years ago and, according to our current standards, all of them had high risk of bias. These uncertainties originate mainly from the lack of methodological information in studies published several decades ago: e.g. the laboratory parameters of the included pregnant women and other potential confounders, like smoking, alcohol consumption, maternal BMI or sex of the infant were usually poorly described. The substantial risk of bias increases the uncertainty of our results and may lead to overestimation or underestimation of the true treatment effect. Differences in supplement form, gestational age, dose or duration may explain the observed heterogeneity of intervention effect; however, the low number of studies included did not allow us to divide them into groups and perform further subgroup analysis. Furthermore, it must be taken into account that the potential reason of the non-significant results of placental weight and length of gestation analysis may be explained by the effect of inadequate sample size. In addition, the studies included in this meta-analysis evaluated approximately 700 birth weights and 400 durations of pregnancy, which would correspond to about 70 SGA birth weights and 40 preterm births in both interventional groups together. Thus this study may be underpowered to make inferences about those most important outcomes.

## Conclusion

Our meta-analysis demonstrated significant dose–response relationship between folate intake and birth weight. However, the results indicated no evidence of any effect of folate supplementation on placental weight and length of gestation. The relative paucity of data that we were able to collect into this systematic review indicates that there is an urgent need to develop further high quality studies focusing on health outcomes of folate supplementation after the first trimester.

## Abbreviations

β: Regression coefficient; CI: Confidence interval; 5-MTHF: 5-methyltetrahydrofolate; NTD: Neural tube defect; RCT: Randomize controlled trial; SE: Standard error; SGA: Small for gestational ag.

## Competing interest

The authors declare that they have no competing interests.

## Authors’ contributions

CB, KF and MT assessed studies for inclusion; CB and MT extracted data, and assessed validity; CD and OWS developed the statistical model; CB and KF conducted meta-analyses. All authors were involved in critical discussion and editing the manuscript for publication, and all authors agreed the final text.

## Source of funding

This research was undertaken as an activity of the European Micronutrient Recommendations Aligned (EURRECA) Network of Excellence ( http://www.eurreca.org) funded by the European Commission (Contract Number FP6 036196–2 (FOOD)).
